# A Multi-Center Study on the Efficacy of Eltrombopag in Management of Refractory Chronic Immune Thrombocytopenia: A Real-Life Experience

**DOI:** 10.4274/tjh.galenos.2018.0307

**Published:** 2019-11-18

**Authors:** Demet Çekdemir, Serkan Güvenç, Füsun Özdemirkıran, Ali Eser, Tayfur Toptaş, Vildan Özkocaman, Handan Haydaroğlu Şahin, Esra Ermiş Turak, Ramazan Esen, Melda Cömert, Sevil Sadri, Müzeyyen Aslaner, Bahar Uncu Ulu, Abdullah Karakuş, Derya Selim Bapur, İnci Alacacıoğlu, Demet Aydın, Atakan Tekinalp, Sinem Namdaroğlu, Funda Ceran, Pınar Tarkun, Demet Kiper, Mustafa Çetiner, Mustafa Yenerel, Ahmet Muzaffer Demir, Güven Yılmaz, Hatice Terzi, Erden Atilla, Ümit Yavuz Malkan, Kadir Acar, Erman Öztürk, Anıl Tombak, Cenk Sunu, Ozan Salim, Nevin Alayvaz, Özkan Sayan, Ülkü Ozan, Mesut Ayer, Zafer Gökgöz, Neslihan Andıç, Ebru Kızılkılıç, Figen Noyan, Mehmet Özen, Funda Pepedil Tanrıkulu, Güçhan Alanoğlu, Hasan Atilla Özkan, Vahap Aslan, Güven Çetin, Alev Akyol Erikçi, Burak Deveci, Fadime Ersoy Dursun, Hasan Dermenci, Pelin Aytan, Mehmet Gündüz, Volkan Karakuş, Can Özlü, Sinan Demircioğlu, Olga Meltem Akay Yanar, Düzgün Özatlı, Levent Ündar, Eyüp Naci Tiftik, Ayhan Gülsan Türköz Sucak, İbrahim Haznedaroğlu, Muhit Özcan, Mehmet Şencan, Murat Tombuloğlu, Gülsüm Özet, Oktay Bilgir, Burhan Turgut, Mehmet Ali Özcan, Kadriye Bahriye Payzın, Mehmet Sönmez, Orhan Ayyıldız, Mehmet Sinan Dal, Şehmus Ertop, Mehmet Turgut, Teoman Soysal, Emin Kaya, Ali Ünal, Mustafa Pehlivan, Işık Atagündüz, Tülin Tuğlular Fıratlı, Güray Saydam, Reyhan Diz Küçükkaya

**Affiliations:** 1 Anadolu Medical Center, Bone Marrow Transplantation Center, Department of Hematology, Kocaeli, Turkey; 2 Yeni Yüzyıl University Gaziosmanpaşa Hospital, Department of Hematology, İstanbul, Turkey; 3 İzmir Atatürk Training and Research Hospital, Clinic of Hematology, İzmir, Turkey; 4 Marmara University Faculty of Medicine, Department of Internal Medicine, Division of Hematology, İstanbul, Turkey; 5 Uludağ University Faculty of Medicine, Department of Internal Medicine, Division of Hematology, Bursa, Turkey; 6 Gaziantep University Faculty of Medicine, Department of Internal Medicine, Division of Hematology, Gaziantep, Turkey; 7 Erciyes University Faculty of Medicine, Department of Internal Medicine, Division of Hematology, Kayseri, Turkey; 8 Van Yüzüncü Yıl University Faculty of Medicine, Department of Internal Medicine, Division of Hematology, Van, Turkey; 9 İnönü University Faculty of Medicine, Department of Internal Medicine, Division of Hematology, Malatya, Turkey; 10 İstanbul University-Cerrahpaşa Cerrahpaşa Faculty of Medicine, Department of Internal Medicine, Division of Hematology, İstanbul, Turkey; 11 Bülent Ecevit University Faculty of Medicine, Department of Internal Medicine, Division of Hematology, Zonguldak, Turkey; 12 Dr. Abdurrahman Yurtaslan Oncology Training and Research Hospital, Clinic of Hematology, Ankara, Turkey; 13 Dicle University Faculty of Medicine, Department of Internal Medicine, Division of Hematology, Diyarbakır, Turkey; 14 Karadeniz Teknik University Faculty of Medicine, Department of Internal Medicine, Division of Hematology, Trabzon, Turkey; 15 Dokuz Eylül University Faculty of Medicine, Department of Internal Medicine, Division of Hematology, İzmir, Turkey; 16 Okmeydanı Training and Research Hospital, Clinic of Hematology, İstanbul, Turkey; 17 Namık Kemal University Faculty of Medicine, Department of Internal Medicine, Division of Hematology, Tekirdağ, Turkey; 18 İzmir Bozyaka Training and Research Hospital, Clinic of Hematology, İzmir, Turkey; 19 Ankara Numune Training and Research Hospital, Clinic of Hematology, Ankara, Turkey; 20 Kocaeli University Faculty of Medicine, Department of Internal Medicine, Division of Hematology, Kocaeli, Turkey; 21 Ege University Faculty of Medicine, Department of Internal Medicine, Division of Hematology, İzmir, Turkey; 22 Koç University Faculty of Medicine, Department of Internal Medicine, Division of Hematology, İstanbul, Turkey; 23 İstanbul University İstanbul Faculty of Medicine, Department of Internal Medicine, Division of Hematology, İstanbul, Turkey; 24 Trakya University Faculty of Medicine, Department of Internal Medicine, Division of Hematology, Edirne, Turkey; 25 Dr. Lütfi Kırdar Kartal Training and Research Hospital, Clinic of Hematology, İstanbul, Turkey; 26 Cumhuriyet University Faculty of Medicine, Department of Internal Medicine, Division of Hematology, Sivas, Turkey; 27 Ankara University Faculty of Medicine, Department of Internal Medicine, Division of Hematology, Ankara, Turkey; 28 Hacettepe University Faculty of Medicine, Department of Internal Medicine, Division of Hematology, Ankara, Turkey; 29 Gazi University Faculty of Medicine, Department of Internal Medicine, Division of Hematology, Ankara, Turkey; 30 Mersin University Faculty of Medicine, Department of Internal Medicine, Division of Hematology, Mersin, Turkey; 31 Sakarya University Faculty of Medicine, Department of Internal Medicine, Division of Hematology, Sakarya, Turkey; 32 Akdeniz University Faculty of Medicine, Department of Internal Medicine, Division of Hematology, Antalya, Turkey; 33 Ondokuz Mayıs University Faculty of Medicine, Department of Internal Medicine, Division of Hematology, Samsun, Turkey; 34 Medicana Çamlıca Hospital, Clinic of Hematology, İstanbul, Turkey; 35 Medical Park Hospital, Clinic of Hematology, Bursa, Turkey; 36 Haseki Training and Research Hospital, Clinic of Hematology, İstanbul, Turkey; 37 Medicana International Ankara Hospital, Clinic of Hematology, Ankara, Turkey; 38 Eskişehir Osmangazi University Faculty of Medicine, Department of Internal Medicine, Division of Hematology, Eskişehir, Turkey; 39 Acıbadem Kozyatağı Hospital, Clinic of Hematology, İstanbul, Turkey; 40 Başkent University İstanbul Hospital, Department of Internal Medicine, Division of Hematology, İstanbul, Turkey; 41 Tokat Gaziosmanpaşa University Faculty of Medicine, Department of Internal Medicine, Division of Hematology, Tokat, Turkey; 42 Dr. Ersin Arslan Training and Research Hospital, Clinic of Hematology, Gaziantep, Turkey; 43 Süleyman Demirel University Faculty of Medicine, Department of Internal Medicine, Division of Hematology, Isparta, Turkey; 44 Yeditepe University Faculty of Medicine, Department of Internal Medicine, Division of Hematology, İstanbul, Turkey; 45 Ümit Hospital, Clinic of Hematology, Eskişehir, Turkey; 46 Bezmialem Vakıf University Faculty of Medicine, Department of Internal Medicine, Division of Hematology, İstanbul, Turkey; 47 İstanbul Yeni Yüzyıl University Gaziosmanpaşa Hospital Faculty of Medicine, Department of Internal Medicine, Division of Hematology, İstanbul, Turkey; 48 Medstar Antalya Hospital, Clinic of Hematology, Antalya, Turkey; 49 Göztepe Training and Research Hospital, Clinic of Hematology, İstanbul, Turkey; 50 Haydarpaşa Numune Training and Research Hospital, Clinic of Hematology, İstanbul, Turkey; 51 Başkent University Training and Research Hospital, Bone Marrow and Stem Cell Transplantation Center, Department of Hematology, Adana, Turkey; 52 Atatürk Training and Research Hospital, Clinic of Hematology, Ankara, Turkey; 53 Muğla Sıtkı Koçman University Training and Research Hospital, Department of Hematology, Muğla, Turkey; 54 Ministry of Health Erzurum Regional Training and Research Hospital, Clinic of Hematology, Erzurum, Turkey; 55 İstanbul University Science Faculty, Department of Molecular Biology and Genetics, İstanbul, Turkey

**Keywords:** Thrombocytopenia, Immune thrombocytopenic, Eltrombopag

## Abstract

**Objective:**

The aim of the present study was to evaluate the efficacy and safety of eltrombopag, an oral thrombopoietin receptor agonist, in patients with chronic immune thrombocytopenia (ITP).

**Materials and Methods:**

A total of 285 chronic ITP patients (187 women, 65.6%; 98 men, 34.4%) followed in 55 centers were enrolled in this retrospective cohort. Response to treatment was assessed according to platelet count (/mm^3^) and defined as complete (platelet count of >100,000/mm^3^), partial (30,000-100,000/mm^3^ or doubling of platelet count after treatment), or unresponsive (<30,000/mm^3^). Clinical findings, descriptive features, response to treatment, and side effects were recorded. Correlations between descriptive, clinical, and hematological parameters were analyzed.

**Results:**

The median age at diagnosis was 43.9±20.6 (range: 3-95) years and the duration of follow-up was 18.0±6.4 (range: 6-28.2) months. Overall response rate was 86.7% (n=247). Complete and partial responses were observed in 182 (63.8%) and 65 (22.8%) patients, respectively. Thirty-eight patients (13.4%) did not respond to eltrombopag treatment. For patients above 60 years old (n=68), overall response rate was 89.7% (n=61), and for those above 80 years old (n=12), overall response rate was 83% (n=10). Considering thrombocyte count before treatment, eltrombopag significantly increased platelet count at the 1^st^, 2^nd^, 3^rd^, 4^th^, and 8^th^ weeks of treatment. As the time required for partial or complete response increased, response to treatment was significantly reduced. The time to reach the maximum platelet levels after treatment was quite variable (1-202 weeks). Notably, the higher the maximum platelet count after eltrombopag treatment, the more likely that side effects would occur. The most common side effects were headache (21.6%), weakness (13.7%), hepatotoxicity (11.8%), and thrombosis (5.9%).

**Conclusion:**

Results of the current study imply that eltrombopag is an effective therapeutic option even in elderly patients with chronic ITP. However, patients must be closely monitored for response and side effects during treatment. Since both response and side effects may be variable throughout the follow-up period, patients should be evaluated dynamically, especially in terms of thrombotic risk factors.

## Abstract (tr)

**Amaç:**

Bu çalışmanın amacı kronik immün trombositopeni (ITP) hastalarında bir oral trombopoietin reseptör agonisti olan eltrombopagın etkinlik ve güvenirliliğini değerlendirmektir.

**Gereç ve Yöntemler:**

Elli beş merkezde izlem altındaki toplam 285 kronik ITP hastası (187 kadın, %65,6) bu geriye dönük küme çalışmasına alınmıştır. Tedaviye yanıt trombosit sayısına göre değerlendirilmiş ve tam yanıt (>100.000/mm^3^), kısmi yanıt (30.000-100.000/mm^3^ veya tedaviden sonra trombosit sayısının bir kat artmış olması) ve yanıtsızlık (<30.000/mm^3^) olarak tanımlanmıştır. Hastaların klinik bulguları, tanımlayıcı özellikleri, tedaviye yanıt ve yan etki bilgileri toplanmış ve aralarındaki ilişki incelenmiştir.

**Bulgular:**

Tanı anında yaş ortalaması 43,9±20,6 (3-95) yıl olan hastalar ortalama 18,0±6,4 (6-28,2) ay izlenmiştir. Tam ve kısmi yanıtı içeren toplam yanıt %86,7 (n=247) bulundu. Sırasıyla 182 (%63,8) ve 65 (%22,8) hastada tam ve parsiyel tedavi yanıtları gözlenmiştir. Otuz sekiz hasta (%13,4) eltrombopag tedavisine yanıt vermemiştir. Altmış yaş üzerindeki hastalarda (n=68) toplam yanıt %89,7 (n=61) bulunurken, bu oran 80 yaş üzerindeki (n=12) hastalarda %83 (n=10) olmuştur. Tedavi öncesi trombosit sayısı göz önüne alındığında, eltrombopag, tedavinin 1., 2., 3., 4. ve 8. haftalarında trombosit sayısını anlamlı şekilde artırmıştır. Kısmi veya tam cevap için gereken süre arttıkça, tedaviye cevap önemli ölçüde azaldığı saptanmıştır. Eltrombopag tedavisinden sonra maksimum trombosit sayısı ne kadar yüksekse, yan etkilerin oluşabilme ihtimalinin o kadar yüksek olabildiği dikkati çekmiştir. En sık görülen yan etkiler baş ağrısı (%21,6), güçsüzlük (%13,7) ve hepatotoksisite (%11,8) ve trombozdur (%5,9).

**Sonuç:**

Mevcut çalışmanın sonuçları, eltrombopag tedavisinin kronik ITP’de, yaşlı hastalar dahil olmak üzere, etkili bir tedavi seçeneği olduğunu göstermektedir. Bununla birlikte, hastalar tedavi sırasında yanıt ve yan etkiler açısından yakından izlenmelidir. Hem cevap hem de yan etkiler, takip süresi boyunca değişken olabileceğinden, hastalar özellikle tromboz risk faktörleri açısından dinamik olarak değerlendirilmelidir.

## Introduction

Immune thrombocytopenia (ITP) is an acquired disorder characterized by a transient or persistent decrease in platelets accompanied with an increased risk of bleeding [1,2,3]. The estimated incidence of ITP is 100 cases per 1 million people annually [4]. Clinical presentation varies in a wide spectrum ranging from asymptomatic or mild cases with bruising and petechiae to severe mucocutaneous bleeding that could be life-threatening [5,6]. Immune thrombocytopenia has been linked to an increased rate of immune-mediated platelet destruction; however, the exact pathophysiological mechanism is still unclear [3].

In chronic ITP, antiplatelet antibodies facilitate platelet destruction and prevent the release of platelets from megakaryocytes, thus resulting in mild to serious thrombocytopenia. Therapeutic strategies for first- or second-line treatment such as corticosteroids, intravenous immunoglobulin, and splenectomy can reduce the destruction of antibody-coated platelets, but the efficacy is limited and serious adverse effects can be seen [7]. Use of immunosuppressive drugs has been restricted because of serious adverse events and splenectomy has been linked to important drawbacks such as infection and thrombosis. Monitoring patients for the effectiveness of the treatment and for side effects is an important issue in the improvement of therapeutic outcomes.

Another treatment strategy is to use thrombopoietin receptor agonists (TPO-RAs) for stimulating platelet production through interaction with the TPO receptors present on megakaryocytes. One such example is eltrombopag, an oral, non-peptide thrombopoietin receptor agonist [8]. Since eltrombopag does not compete with endogenous TPO binding at the extracellular TPO-R domain, it may possess an additive effect to thrombopoietin [9]. As a consequence, the Janus kinase/signal transducer and activator of transcription (JAK/STAT) signaling pathway stimulates megakaryocytopoiesis, while autoantibody generation is not detected [10]. Furthermore, eltrombopag does not influence agonist-induced platelet aggregation or activation [1]. Eltrombopag produces a quick and sustainable increase in platelet counts and is generally well tolerated in patients with chronic ITP.

The present study aimed to analyze the outcomes of eltrombopag treatment in patients with chronic ITP in clinical practice in Turkey and to estimate the demographic, clinical, and hematological variables that may have implications for therapeutic response.

## Materials and Methods

### Patients and Study Design

This retrospective study (2011-2017) was conducted in 55 tertiary care centers of Turkey. Data were collected from medical files of 285 chronic ITP patients, of whom 187 were women (65.6%) and 98 were men (34.4%). Patients with a diagnosis of chronic ITP according to the international consensus report [11] irrespective of their age at diagnosis were eligible for inclusion if they received eltrombopag at any time in their treatment schedule. The exclusion criteria for treatment with eltrombopag consisted of HIV, hepatitis B, or hepatitis C infections; cardiovascular diseases; malignancy; chemotherapy or radiotherapy; prior diagnosis of myelodysplastic syndrome or aplastic anemia; and presence of two or more risk factors for thrombosis, such as smoking, diabetes mellitus, hypercholesterolemia, or hereditary thrombophilic disorders. Patients with a history of thrombosis were also excluded from the study because of the contraindication for these patients as a policy of the Ministry Health of Turkey.

The study was performed in accordance with the Declaration of Helsinki and conducted after the approval of the local institutional review board. Written informed consent was provided for all patients enrolled in this study. Chronic ITP is defined as persistent thrombocytopenia despite conventional initial management [12]. Eltrombopag was administered at doses of 50 mg as a starting dose as approved by the Turkish Ministry of Health. After 2 weeks of treatment, if the platelet levels were <30,000/µL, the dose was increased up to a maximal daily dose of 75 mg in increments of 25 mg. Achieving a platelet level between 150,000/µL and ≤250,000/µL, the daily dose was tapered by 25 mg. If platelet levels reached above 250,000/µL, eltrombopag was stopped, and after decreasing to <100,000/µL the treatment was restarted by reducing the last daily dose by 25 mg. Descriptive data (age at diagnosis of chronic ITP, sex), therapeutic response (none, partial, or complete), side effects (absent or present), and severity of findings linked with bleeding (none, mild, moderate, severe, or life-threatening) were recorded. Platelet counts at first admission, before treatment, and at the 1^st^, 2^nd^, 3^rd^, 4^th^, and 8^th^ weeks; the number of days with platelet count of >30,000/µL; maximum platelet counts after treatment; time to reach maximum platelet counts after treatment; and duration of follow-up were documented. Correlations between these descriptive, clinical, and hematological parameters were analyzed.

Severe or life-threatening bleeding was defined as either intracranial hemorrhage or bleeding that caused hemodynamic compromise and required intervention. Moderate bleeding was defined as bleeding that required blood transfusion but did not result in hemodynamic compromise. Minor bleeding was defined as bleeding that did not meet the criteria for either severe or moderate bleeding.

Response to treatment was defined as none (platelet count <30,000/µL), partial (platelet count between 30,000/µL and 100,000/µL or platelet count double the initial value), or complete (platelet count >100,000/µL).

### Statistical Analysis

Data were analyzed using IBM SPSS 20.0 software for Windows (IBM Corp., Armonk, NY, USA). The normal distribution of continuous variables was evaluated with the Kolmogorov-Smirnov test. Parametric tests were used for variables distributed normally, while non-parametric tests were utilized for variables without normal distribution. Correlation between variables was tested with Spearman’s rho test. Categorical variables were compared with Pearson chi-square and Fisher exact tests, while two independent groups were compared using t-tests and Mann-Whitney U tests. Quantitative variables are demonstrated as mean ± standard deviation or median-interquartile range. The confidence interval was 95% and differences associated with a p-value of less than 0.05 were considered as statistically significant.

## Results

The average age at diagnosis was 43.9±20.6 (range: 3 to 95) years. An outline of the demographic and clinical data of the present series is shown in Table 1. Before starting the eltrombopag treatment, clinical findings associated with bleeding were as follows: mild bleeding in 110 (38.6%) patients, moderate in 78 (27.4%), severe or life-threatening in 20 (7%), and no bleeding in 77 (27%). The numbers of chronic ITP patients with no response, partial response, or complete therapeutic response to eltrombopag treatment were 38 (13.4%), 65 (22.8%), and 182 (63.8%), respectively. Using a platelet level cut-off of >30,000/µL, overall response rate was 86.7% (n=247). Considering patients above 60 years old (n=68), overall response rate was 89.7% (n=61), and above 80 years old (n=12), overall response rate was 83% (n=10). The findings of the older patients above 60 and 80 years are listed in Table 2.

Platelet counts at first admission and before and after treatment (1^st^, 2^nd^, 3^rd^, 4^th^, and 8^th^ weeks) as well as maximum platelet count, number of days with platelet count >30,000/µL, and interval (weeks) needed to achieve maximal platelet counts are presented in Table 3.

The median number of days required to achieve a platelet count of >30,000/µL was 14 (range: 3-210). Median maximal platelet counts were 275,000-346,000/µL (range: 5150 to 2,068,000) and time interval until achievement of maximal platelet count was 8-18 weeks (range: 1-202).

Notably, there was a significant positive correlation between treatment response and number of days to achieve platelet count of >30,000/µL (p=0.009, r=0.180). In contrast, age (p=0.129, r=0.764), platelet count at diagnosis (p=0.764, r=-0.020), and maximum platelet count after eltrombopag treatment (p=0.133, r=0.107) did not exhibit any correlation with treatment response.

Correlation analysis demonstrated that the higher the maximum platelet count was after eltrombopag treatment, the more likely side effects were to occur (p=0.004, r=0.215). Table 4 demonstrates the results of correlation analysis seeking the association between clinical variables, platelet counts.

Sex (p=0.594) and age (≤40 years and >40 years) (p=0.218) did not have a remarkable effect on treatment response. Similarly, platelet count at diagnosis did not seem to have a significant impact on treatment response (p=0.214).

Patients with platelet count of >30,000/µL in the 1^st^, 2^nd^, 3^rd^, 4^th^, and 8^th^ weeks after eltrombopag treatment exhibited a better response to treatment (p>0.001 for all). Pearson chi-square test results indicated that treatment response was similar among patients who had any degree of bleeding (p=0.089). Treatment response was statistically significantly associated with number of days with platelet count of >30,000/µL (p=0.010), maximal platelet count (p<0.001), and duration of follow-up (p<0.001). On the contrary, treatment response was not affected by the week in which the highest platelet count was observed (p=0.121).

Our results demonstrated that the occurrence of side effects was not affected by sex (p=0.079), age (≤40 years and >40 years) (p=0.079), or platelet count at diagnosis (p=0.586) or in the 1^st^ week (p=0.636), 2^nd^ week (p=0.761), 3^rd^ week (p=0.850), 4^th^ week (p=0.485), and 8^th^ week (p=0.527) after eltrombopag treatment. No association was noted between occurrence of side effects and number of days with platelet count of >50,000/µL (p=0.206), the week in which maximal platelet count was achieved (p=0.231), or duration of follow-up (p=0.685).

Side effects were observed in 62 (21.8%) cases (Table 1). The most common side effects were headache (21.6%), weakness (13.7%), hepatotoxicity (11.8%), venous thrombosis (4.2%), and arterial thrombosis (1.7%). Itching, erythromelalgia, transient ischemic attack, myalgia, and neuropathy were observed in 2 patients (3.9%) each.

The overall thrombosis rate including arterial (n=5) and venous thrombosis (n=12) was 5.9%. Thromboses presented clinically mostly as deep vein thrombosis. Pulmonary embolism was recorded in 3 patients. For arterial thrombosis, the main presentation was a transient ischemic attack (n=3). One patient suffered from ischemic stroke and one patient suffered from sudden death clinically attributed to a cardiac event. The thrombosis rate was found to be 2% in patients over 60 years of age and 16% in patients over 80 years of age. Clinical features and management of patients with thrombosis are summarized in Table 5.

Other side effects observed in only one patient each were as follows: hair loss, maculation, thrombocytosis, erythrocytosis, frequent tonsillitis, frequent pneumonia, diarrhea, and ileus. Side effects of any kind of grades 3-4, mainly thromboembolic events, were found at a rate of 6.3%.

## Discussion

The present study was performed to investigate the variables that may be associated with treatment response and side effects after eltrombopag treatment for chronic ITP in daily practice. The overall response to eltrombopag in this cohort was 86.3%. This finding is consistent with previous prospective and retrospective studies [13,14,15]. Our data have shown that platelet counts before, during, and after treatment as well as maximal platelet counts, duration of follow-up, and number of days to achieve platelet count of >30,000/µL could have predictive potential for therapeutic response. Side effects were found to be significantly more common in patients with higher platelet counts after treatment. In our ITP cohort, the time to reach maximum platelet levels ​​during treatment with eltrombopag was quite variable (1-202 weeks). Platelet counts during different periods in the course of eltrombopag treatment for chronic ITP may possess important implications in terms of therapeutic response and side effect profile. In general, the response to treatment and side effects were similar in the elderly population, whereas thrombosis was more common in patients over 80 years of age, although the number of cases was small.

In chronic ITP, the goal of treatment is to provide sufficient platelet levels to avoid major bleeding and to minimize treatment-related toxicity. Patients with platelet counts of ≥30,000/µL are supposed to have adequate hemostasis and generally do not require treatment in the absence of a history of bleeding [12]. Patients with ITP who have platelet counts above the normal minimum-maximum may have a risk of thrombotic or thromboembolic complications [16]. Efforts must be made to improve functional capacity and maturation of platelets as well as platelet count to overcome bleeding problems in patients with chronic ITP while decreasing the side effects, especially serious thrombotic complications.

Our results suggest that platelet counts obtained at different intervals in the course of eltrombopag treatment can serve as important predictors for treatment response and occurrence of side effects. Patients with insufficient responses to treatments such as corticosteroids, immunoglobulins, or rituximab may also be appropriate candidates for eltrombopag treatment. Regular platelet counts and close follow-up are mandatory for monitoring the effectivity of treatment and potential safety issues.

Patients in eltrombopag clinical trials experienced both arterial and venous thrombosis. Of 135 patients receiving eltrombopag in the RAISE study, three (2.2%) developed venous thrombosis [8]. The EXTEND extension study followed 299 patients for up to 5 years and reported nine patients with venous thrombosis and seven patients with arterial thrombosis (5.4%) [17]. In the present study, venous thrombosis was observed in 12 patients (5.9%) and arterial thrombosis in 5 patients (1.7%). Although eltrombopag was generally well tolerated during treatment in RAISE, transient increases of alanine aminotransferase and indirect bilirubin concentrations were reported, perhaps related to the metabolism of both eltrombopag and bilirubin by UGT1A1 [8]. All aminotransferase abnormalities were resolved; however, aminotransferase and bilirubin levels must be monitored before initiation of and during eltrombopag treatment, and treatment should be stopped if necessary. In the present study, none of the patients experienced increases in liver tests that required permanently discontinuing the drug. Of 135 patients in the RAISE study, 30% experienced headaches and 10% experienced fatigue, while in the present study, 4.2% of the study group reported headaches and 1.8% reported fatigue. The patient who died suddenly during follow-up had normal platelets at the last visit and the exact cause of death was clinically attributed to a cardiac event.

The main limitations of the current trial include the retrospective design, lack of a control group, and possible impacts of social, genetic, environmental, metabolic, and ethnic factors on treatment outcomes and side effects.

## Conclusion

The results of the current study indicate that eltrombopag can be a safe and effective therapeutic option in refractory and chronic ITP, even in older populations. However, patients must be closely monitored for therapeutic response and side effects during treatment. Since both responses and side effects may be variable throughout the follow-up period, patients should be evaluated dynamically, especially in terms of thrombosis risk factors.

## Figures and Tables

**Table 1:**
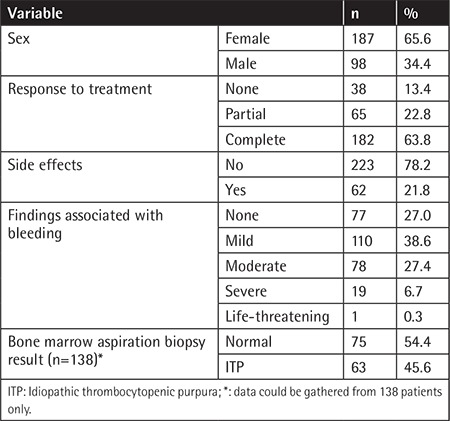
Descriptive and clinical data (n=285).

**Table 2:**
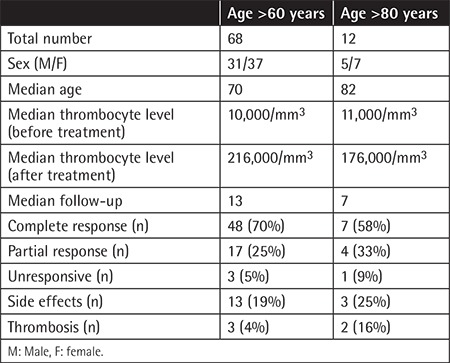
Results of the older population.

**Table 3:**
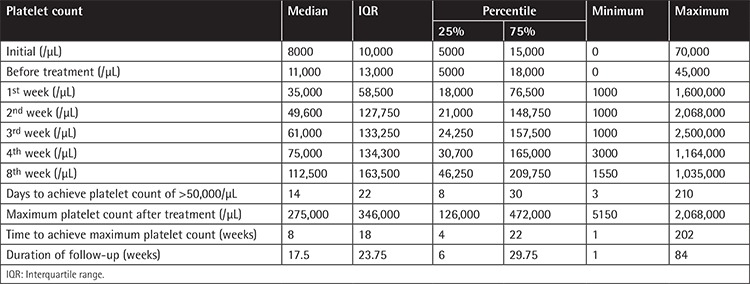
Data related to platelet count during the course of eltrombopag treatment.

**Table 4:**
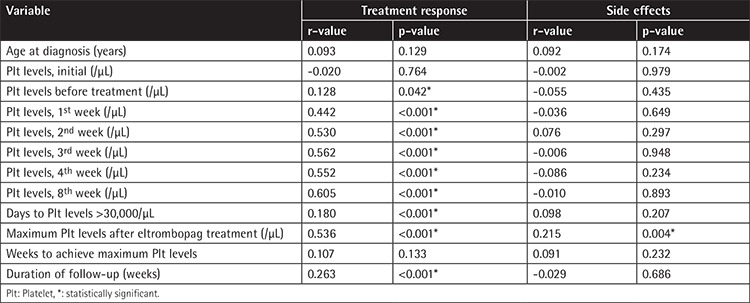
Correlations between clinical variables, platelet counts, treatment response, and side effects.

**Table 5:**
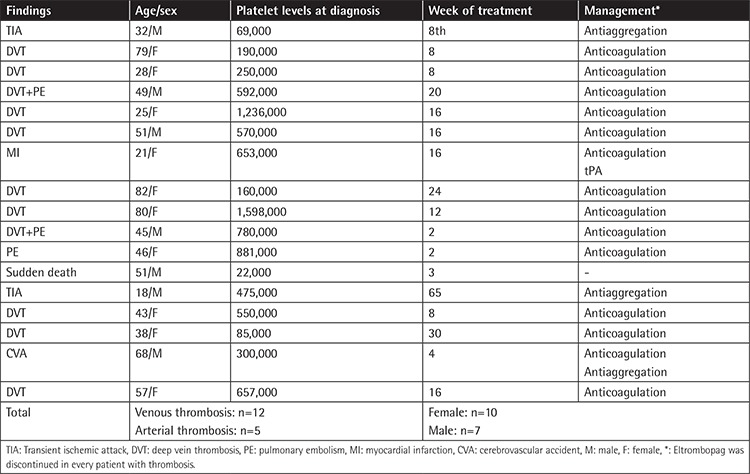
Characteristic features of patients with thrombosis.

## References

[1] Kühne T, Imbach P (2010). Eltrombopag: an update on the novel, non-peptide thrombopoietin receptor agonist for the treatment of immune thrombocytopenia. Ann Hematol.

[2] Chang M, Nakagawa PA, Williams SA, Schwartz MR, Imfeld KL, Buzby JS, Nugent DJ (2003). Immune thrombocytopenic purpura (ITP) plasma and purified ITP monoclonal autoantibodies inhibit megakaryocytopoiesis in vitro. Blood.

[3] Cooper N, Bussel J (2006). The pathogenesis of immune thrombocytopaenic purpura. Br J Haematol.

[4] Neylon AJ, Saunders PW, Howard MR, Proctor SJ, Taylor PR (2003). Clinically significant newly presenting autoimmune thrombocytopenic purpura in adults: a prospective study of a population-based cohort of 245 patients. Br J Haematol.

[5] Stasi R, Provan D (2004). Management of immune thrombocytopenic purpura in adults. Mayo Clin Proc.

[6] Cines D, Bussel J (2005). How I treat idiopathic thrombocytopenic purpura (ITP). Blood.

[7] Çekdemir D, Diz Küçükkaya R (2014). Treatment and prognosis of immune thrombocytopenia. Turkiye Klinikleri J Hematol-Special Topics.

[8] Cheng G, Saleh MN, Marcher C, Vasey S, Mayer B, Aivado M, Arning M, Stone NL, Bussel JB (2011). Eltrombopag for management of chronic immune thrombocytopenia (RAISE): a 6-month, randomised, phase 3 study. Lancet.

[9] Grainger JD, Locatelli F, Chotsampancharoen T, Donyush E, Pongtanakul B, Komvilaisak P, Sosothikul D, Drelichman G, Sirachainan N, Holzhauer S, Lebedev V, Lemons R, Pospisilova D, Ramenghi U, Bussel JB, Bakshi KK, Iyengar M, Chan GW, Chagin KD, Theodore D, Marcello LM, Bailey CK (2015). Eltrombopag for children with chronic immune thrombocytopenia (PETIT2): a randomised, multicentre, placebo-controlled trial. Lancet.

[10] Erickson-Miller CL, DeLorme E, Tian SS, Hopson CB, Stark K, Giampa L, Valoret EI, Duffy KJ, Luengo JL, Rosen J, Miller SG, Dillon SB, Lamb P (2005). Discovery and characterization of a selective, nonpeptidyl thrombopoietin receptor agonist. Exp Hematol.

[11] Provan D, Stasi R, Newland AC, Blanchette VS, Bolton-Maggs P, Bussel JB, Chong BH, Cines DB, Gernsheimer TB, Godeau B, Grainger J, Greer I, Hunt BJ, Imbach PA, Lyons G, McMillan R, Rodeghiero F, Sanz MA, Tarantino M, Watson S, Young J, Kuter DJ (2010). International consensus report on the investigation and management of primary immune thrombocytopenia. Blood.

[12] Chouhan JD, Herrington JD (2010). Treatment options for chronic refractory idiopathic thrombocytopenic purpura in adults: focus on romiplostim and eltrombopag. Pharmacotherapy.

[13] González-López TJ, Fernández-Fuertes F, Hernández-Rivas JA, Sánchez-González B, Martínez-Robles V, Alvarez-Román MT, Pérez-Rus G, Pascual C, Bernat S, Arrieta-Cerdán E, Aguilar C, Bárez A, Peñarrubia MJ, Olivera P, Fernández-Rodríguez A, de Cabo E, García-Frade LJ, González-Porras JR (2017). Efficacy and safety of eltrombopag in persistent and newly diagnosed ITP in clinical practice. Int J Hematol.

[14] Mazza P, Minoia C, Melpignano A, Polimeno G, Cascavilla N, Di Renzo N, Specchia G (2017). The use of thrombopoietin-receptor agonists (TPO-RAs) in immune thrombocytopenia (ITP): a “real life” retrospective multicenter experience of the Rete Ematologica Pugliese (REP). Ann Hematol.

[15] Wong RSM, Saleh MN, Khelif A, Salama A, Portella MSO, Burgess P, Bussel JB (2017). Safety and efficacy of long-term treatment of chronic/persistent ITP with eltrombopag: final results of the EXTEND study. Blood.

[16] Garnock-Jones KP (2011). Eltrombopag: a review of its use in treatment-refractory chronic primary immune thrombocytopenia. Drugs.

[17] Saleh MN, Bussel JB, Cheng G, Meyer O, Bailey CK, Arning M, Brainsky A;, EXTEND Study Group (2013). Safety and efficacy of eltrombopag for treatment of chronic immune thrombocytopenia: results of the long-term, open-label EXTEND study. Blood.

